# Manipulation of phase slips in carbon-nanotube-templated niobium-nitride superconducting nanowires under microwave radiation

**DOI:** 10.1038/s41598-020-71218-0

**Published:** 2020-08-31

**Authors:** Kota Kato, Tasuku Takagi, Takasumi Tanabe, Satoshi Moriyama, Yoshifumi Morita, Hideyuki Maki

**Affiliations:** 1 Department of Applied Physics and Physico-Informatics, Faculty of Science and Technology, Keio University, Hiyoshi, Yokohama, 223-8522 Japan; 2 Department of Electronics and Electrical Engineering, Faculty of Science and Technology, Keio University, Hiyoshi, Yokohama, 223-8522 Japan; 3 International Center for Materials Nanoarchitectonics (WPI-MANA), National Institute for Materials Science (NIMS), Namiki, Tsukuba, Ibaraki 305-0044 Japan; 4 Faculty of Engineering, Gunma University, Kiryu, Gunma 376-8515 Japan; 5 Center for Spintronics Research Network, Keio University, Yokohama, 223-8522 Japan

**Keywords:** Nanoscience and technology, Nanoscale devices, Electronics, photonics and device physics, Superconducting devices

## Abstract

We study the manipulation of thermal/quantum phase slips (tPSs/qPSs) in ultra-thin niobium-nitride superconducting nanowires (scNW) grown on carbon-nanotube templates. These NWs exhibit resistive steps in current–voltage (*I*–*V*) characteristics, and the number of phase slip centers (PSCs) in an NW can be tuned by the NW length. Under microwave (MW) radiation, emergence of each single PSC can be precisely controlled by varying the MW power. For thin and short scNW, a dip structure between the qPS-dominated low-temperature region and the tPS-dominated high-temperature region were observed owing to anti-proximity effect by electrodes.

Superconducting nanowires (scNWs)^1,2^ have been attracting interest in the context of modern nanotechnology because of their superconducting properties unique to one-dimensional system. For quantum-device applications, single-photon detectors^3–6^, qubits^7–10^, and quantum current standards^11^ are promising devices for quantum computing, cryptography, and metrology. In scNW, the quantum or thermal fluctuations give rise to phase slips (PSs)^12,13^. The thermal activation of PSs (tPSs) is dominant at high temperatures^13,14^, and as the temperature is lowered, quantum PSs (qPSs) take place, which are a type of macroscopic quantum tunneling^15–20^. Under microwaves (MWs), moreover, a dynamical resistive state, i.e., PS centers (PSCs), emerges^21–23^. Manipulation of PSCs with such external perturbations has been recently demonstrated^24,25^.

Here, we investigate the manipulation of tPS and qPS under MWs in ultra-thin scNW grown on carbon-nanotube (CNT) templates. From the resistive steps due to PSC in current–voltage (*I*–*V*) characteristics, we found that the number of PSCs in single NW can be tuned by the NW length. We also found that superconducting and PSC states can be switched by MWs and the emergence of each single PSC can be precisely controlled by varying the MW power. In resistance–temperature (*R*–*T*) characteristics, moreover, a clear dip structure between the qPS-dominated low-temperature region and the tPS-dominated high-temperature region can be observed owing to strong anti-proximity effect (APE) for the thin and short scNW.

We fabricated niobium-nitride scNWs (NbN-scNW) based on suspended CNT^17,19,20,26–29^. Figure 1a shows the schematics of our NbN-scNW device. The details of the fabrication are given in Ref.^29^. In this paper, we employ three scNWs, which we call SscNW, LscNW, and TscNW (short scNW, long scNW, and thin scNW, respectively). We exposed two scNWs to MW radiation, which are SscNW (*L* = 0.55 μm) and LscNW (*L* = 3.1 μm) with the width *W* = 35 nm for both. We also studied thin and short scNW (TscNW) without MW radiation with *L* = 0.75 μm and *W* = 26 nm for a comparative study. Figure 1b, c show SEM images of SscNW and LscNW on suspended CNTs, which lead to the estimation of *L* and *W*. All the samples are wired in a four-probe configuration, and their electrical transport properties are measured in a pumped helium cryostat (see also Supplemental Material text and Supplemental Fig. S1). The MW radiation is shown in Fig. 1a schematically. The MW is fed through a coaxial MW line, which is weakly coupled to the device. Here a comment is in order for our sample quality. As was done in our previous paper^29^, our sample quality was reconfirmed by the transmission electron microscope (TEM) image, which implies the roughness of our scNW is comparable with previous studies (e.g. about few nanometers^5^).

**Figure 1:**
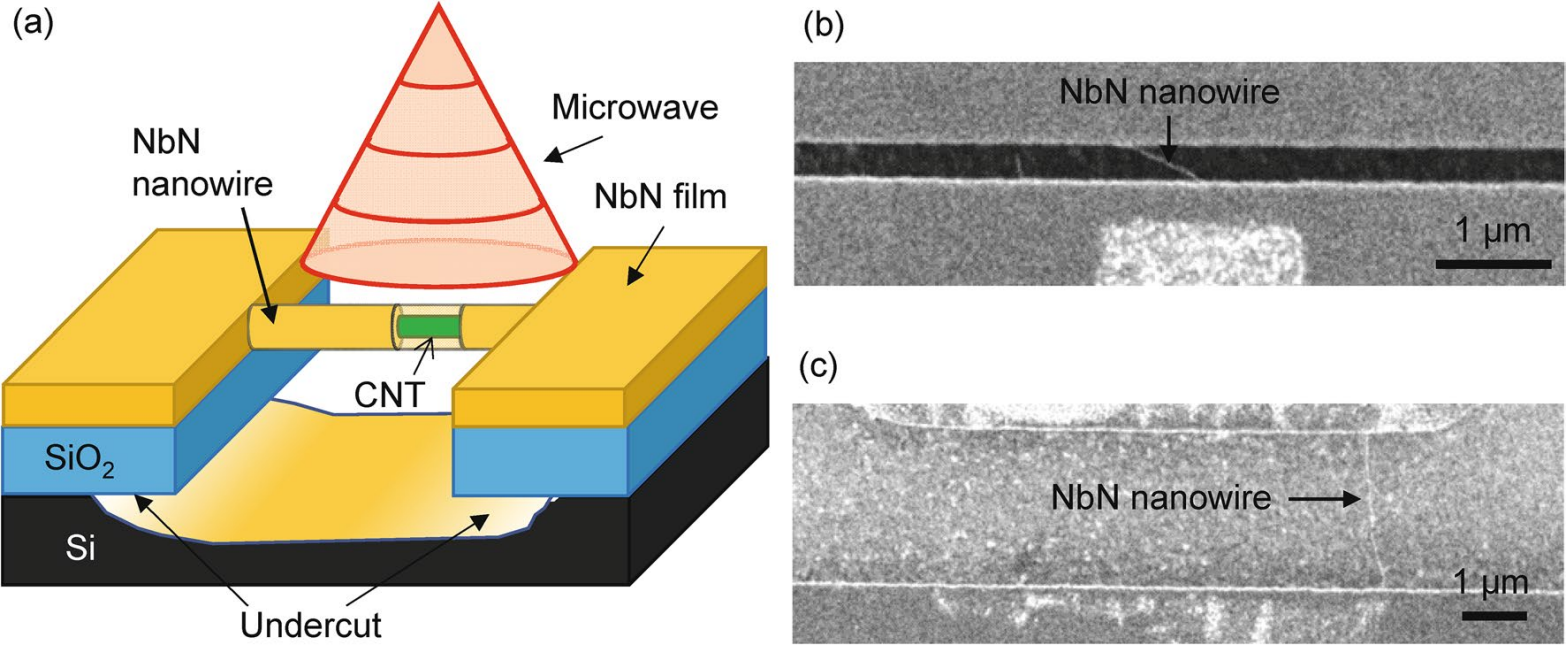
Fabricated NbN-scNW devices. (**a**) Schematic of our NbN-scNW device based on the CNT template. The nanowire and the electrodes are simultaneously sputtered and seamlessly connected to each other. The undercut structure completely surrounds the circumference of the electrodes, including the bonding pads, leading to electrical disconnection of the electrodes except through the nanowire. scNW is exposed to the MW radiation as shown schematically. (**b**) SEM image of SscNW (width *W* = 35 nm, length *L* = 0.55 μm) and (**c**) SEM image of LscNW (*W* = 35 nm, *L* = 3.1 μm).

Figure 2a presents a mapping of the differential resistance d*V*/d*I* for the SscNW as a function of the bias current *I* and the temperature *T*. The white broken curve in Fig. 2a denotes the Bardeen’s formula for the critical pair-breaking current^30^, which is consistent with the temperature dependence of the switching current in the low-temperature regime. The asymmetry in Fig. 2a indicates that the *I*–*V* curve has hysteresis, which has been reported in several scNWs and is attributed to Joule heating and/or an analog of the hysteresis in the resistively and capacitively shunted junction model^13,26,29,31,32^.

**Figure 2:**
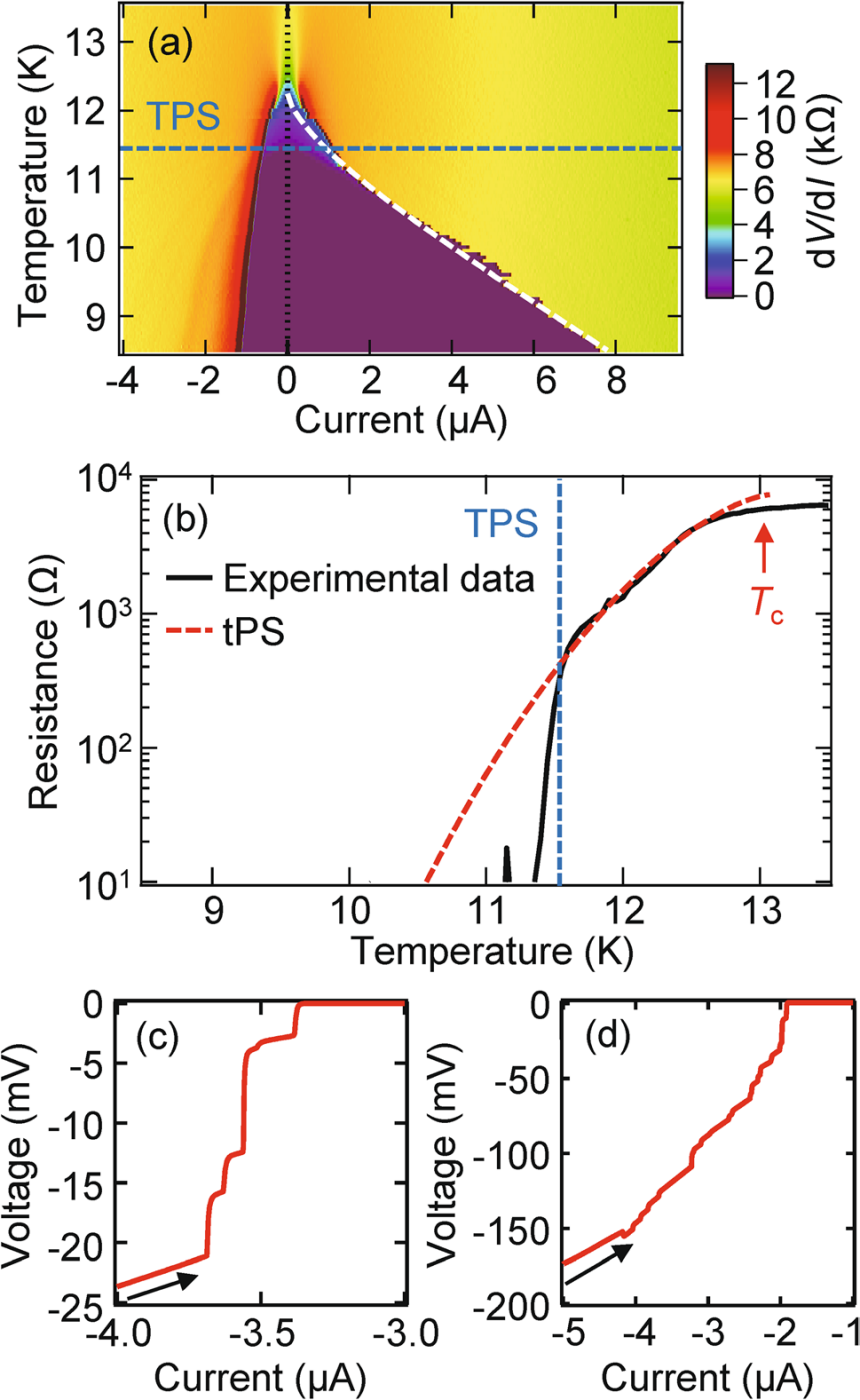
Electric and phase slip properties of SscNW. (**a**) Plot of the differential resistance, d*V*/d*I*, for SscNW as a function of the (increasing) bias current and temperature. The asymmetry indicates that the *I–V* curve has hysteresis. As shown in the white broken curve, in the low-temperature regime, the temperature dependence of the switching current closely matches Bardeen’s formula for the critical pair-breaking current. (**b**) *R*–*T* characteristic curves for SscNW. The red broken line is a fitting to the formula of tPS resistance *R*_tPS_. *I*–*V* characteristics for (**c**) SscNW and (**d**) LscNW at *T* = 1.65 K. Resistive steps show the emergence of PSCs.

The temperature dependence of resistance is shown in Fig. 2b. PSs become evident away from the critical-fluctuation regime below *T*_c_. PS causes a finite resistance in superconductors, and the superconducting order parameter becomes zero somewhere along the wire, which leads to a relative PS of 2π at some rate and a voltage drop. According to Little’s model^13,29,32^, residual resistance arising from thermally activated PS (tPS) below *T*_c_ is given by the following equation:

1 $$R_{{{\text{tPS}}}} \left( T \right) = R_{{\text{n}}} \exp \left( { - \frac{{{\Delta }F\left( T \right)}}{{k_{{\text{B}}} T}}} \right),$$

where $\Delta F\left( T \right) = 0.83k_{{\text{B}}} T_{\text{c}} \left( {R_{q} /R_{{\text{n}}} } \right)\left( {L/\xi \left( 0 \right)} \right)\left( {1 - T/T_{\text{c}} } \right)^{3/2}$ is the superconducting condensation energy (i.e., the energy barrier for PS), $R_{q} = h/4e^{2} = 6.45\,{\text{k}}$Ω is the quantum resistance for Cooper pairs, $\xi \left( 0 \right)$ is the GL coherence length at 0 K, and $k_{{\text{B}}}$ is the Boltzmann constant. The broken curve in Fig. 2b shows a fit of this model to the data. The tPS fitting curve is obtained with $L = 0.55\; \upmu {\text{m}},R_{\text{n}} = 7.8\;{\text{k}}$Ω, and two fitting parameters, i.e., $T_{{\text{c}}} = 13\; {\text{K and }}\xi \left( 0 \right) = 4.7\; {\text{nm}}$. This value of $\xi \left( 0 \right)$ is consistent with that in previous reports ($\xi \left( 0 \right) \approx 5\; {\text{nm}}$). The $T_{{\text{c}}}$ is generally higher than the transition temperature of the 2D film^29^, which is here 11.5 K. Although it is well described by the tPS picture in the temperature range down to approximately 11.5 K, our experimental data deviate from the tPS model at lower temperatures (i.e., below 11.5 K), which should be due to dimensional crossover in thicker scNWs. In a thicker scNW, generally speaking, a crossover can take place from 1D to higher dimensions. Such a dimensional crossover has been observed in several quasi-1D superconductors e.g. Ref.^33^. Instead of t and/or qPS behavior, in the low-temperature limit of such systems, drop to zero resistance can be observed due to crossover from 1D to higher dimensions. We note that these SscNW and LscNW exhibit no qPS behavior because of thicker scNW in contrast to the thinner scNW in Ref.^29^ and TscNW discussed later. As shown in Fig. 2a, the hysteresis fades out above ~ 11.5 K (the blue broken line), where the thermal vortices are deconfined and heating due to residual resistance/thermal fluctuations is enhanced.

Figure 2c,d show the *I–V* characteristic curves for SscNW and LscNW, respectively. A high bias current induces a set of discrete resistance steps, which should be identified with PSCs due to sample inhomogeneities^13,14,29,34,35^. Moreover, comparing the number of PSCs in SscNW and LscNW, we found that it increases in the longer scNW, indicating that the number of PSCs in one NW can be controlled by the NW length (see Fig. 2c,d). The length dependence of the number of PSCs is in reasonable agreement with the theoretical calculation^14^ by the Skocpol, Beasley, and Tinkham (SBT) model^34,35^.

We expose the SscNW to MW radiation. A typical *I*–*V* characteristic, *V*(*I*), of our SscNW is shown in Fig. 3a. As shown in Fig. 2a, *V*(*I*) exhibits a hysteretic behavior at low temperatures without MWs, whereas the asymmetry due to hysteresis fades out at high temperatures. Generally, the superconducting state is switched into the normal state at a “switching current” as the current is increased. Then, after becoming the Joule-heated normal state and decreasing the bias current, the state is returned to the SC state again at a “return/retrapping” current. When MWs are applied, the switching current and its stochastic nature are suppressed, the return/retrapping current is basically kept, and the hysteretic behavior fades out. The hysteretic behavior quickly disappears at a certain power, *P*_c_ (~ − 3.8 dBm in Fig. 3a,b). Note that the switching current is basically “stochastic” because of the nucleation of a few or single PS, whereas the return/retrapping current is “deterministic”^23^. The absence of Shapiro steps implies the absence of phase coherence to synchronize with applied MWs.

**Figure 3:**
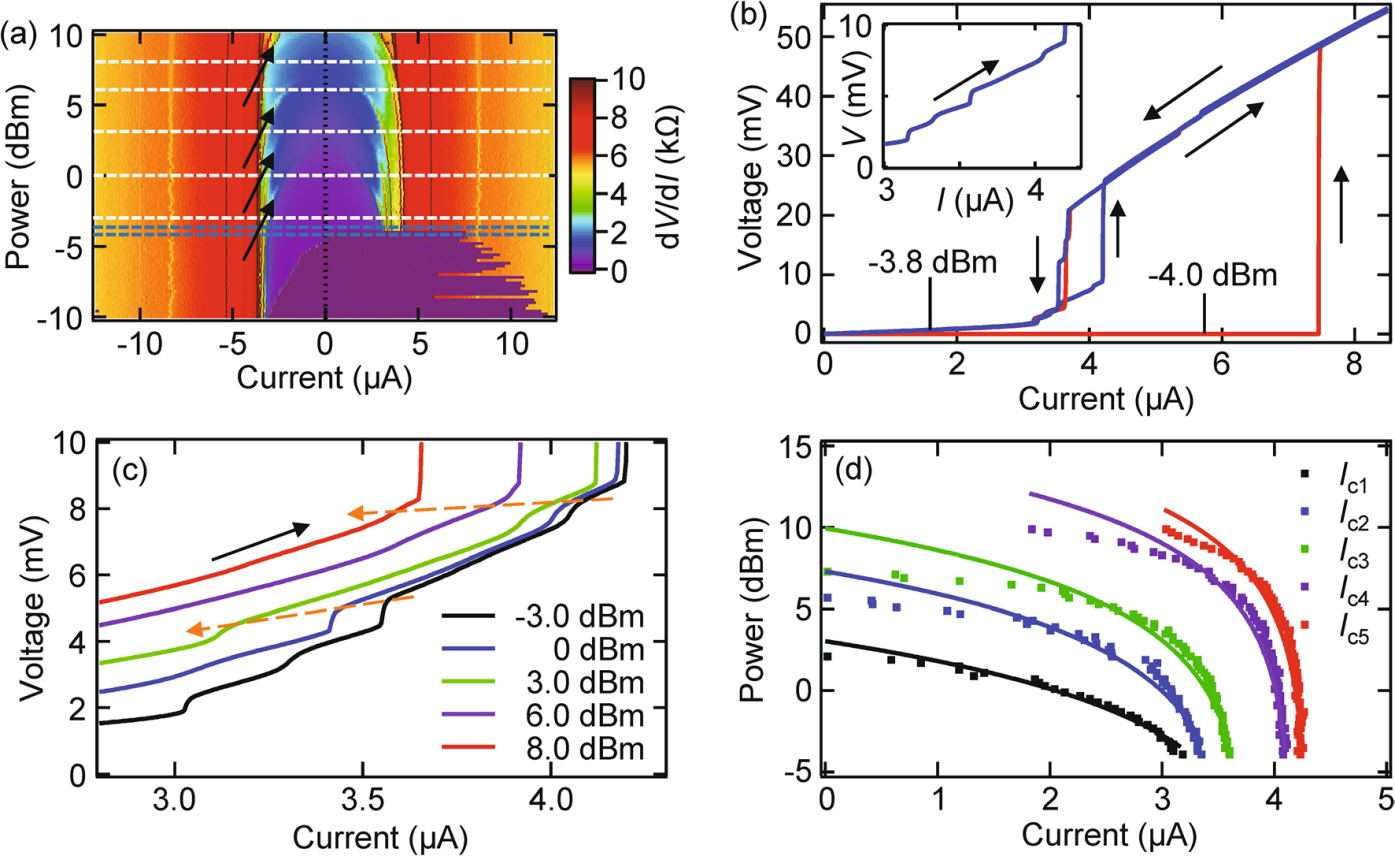
PSC control by MW irradiation in SscNW. (**a**) Mapping of d*V*/d*I* for SscNW as a function of the bias current *I* and MW power *P* at the MW frequency *f* = 26.57 GHz and the temperature *T* = 1.65 K. Blue broken lines indicate MW powers of *P* =  − 4.0, − 3.8 dBm used in (**b**). Arrows indicate the resistive steps due to the emergence of PSCs. (**b**) *I*–*V* characteristics for SscNW at *P* =  − 4.0, − 3.8 dBm (i.e., just below and above *P*_c_) indicated by blue broken lines in (**a**). Inset: a close-up of the *I*–*V* characteristics. (**c**) *I*–*V* characteristics as above for SscNW at *T* = 1.65 K and *P* =  − 3.0, 0, 3.0, 6.0, 8.0 dBm indicated by white broken lines for (**a**). Dashed arrows correspond to the movement of *I*_c_ in (**d**). (**d**) “Critical” current *I*_c_ curves, which are the voltage steps in (**a**) due to the emergence of PSC. Phenomenologically, *I*_c_ curves are assumed to be *I*_c_(*P*) = *I*_0_ − *AP*, where *I*_0_ is the critical current without applied MWs and *A* is the coefficient.

As high-power MWs are applied, MW-assisted PSCs take place in the sweeping up current (see Fig. 3b inset and Fig. 3c). At high power, the switching current is reduced and the wire switches into PSC state at a low bias current. Here, Joule heating dissipation in the wire is weak enough not to heat the wire above *T*_c_ in this regime, which leads to the nucleation of PSCs near the switching current, i.e., the emergence of MW-assisted PSCs^21–23^. In Fig. 3a, we can clearly observe the resistive steps (arrows in Fig. 3a) due to the emergence of the PSCs and can trace them as a function of MW power (see Fig. 3a), which is distinguished from simple heating effects. This indicates that the emergence of every single PSC can be precisely controlled by changing the MW power, which has higher controllability compared with the simple temperature change.

Figure 3d shows a “critical” current *I*_c_ at which the resistive steps occur because of the emergence of PSCs. Phenomenologically, *I*_c_ is assumed to be following equation:

2 $$I_{{\text{c}}} \left( P \right) = I_{0} - AP,$$

where *P* is the MW power, *I*_0_ is the critical current without applied MWs, and *A* is an adjustable parameter. The solid lines in Fig. 3d show a fit of this formula to the experimental data. Since *I*_c_(*P*) is proportional to the MW power, it is found that these are correlated to heating by MW irradiation.

We exposed the LscNW to MW radiation. A typical *I*–*V* characteristic, *V*(*I*), of our LscNW is shown in Fig. 4a, which is basically the same as for the SscNW in the above section except many resistive steps due to many PSCs in a long scNW. In this LscNW, moreover, it is to be noted that “excess voltage” is observed (Fig. 4b)^36–38^. Near the PSC, the (Bogoliubov) quasiparticle diffuses the distance $\Lambda_{{\text{Q}}}$ away from the PSC. Such an excess voltage has only been observed when the distance between voltage probes is narrow within the quasiparticle diffusion length $\Lambda_{{\text{Q}}}$^37^. This is not the case in our study, as discussed next, and further theoretical study is needed. The frequency dependence of *R*_PSC_ is shown in Fig. 4c, where *R*_PSC_ is the differential resistance of the PSC slightly above *P*_c_, as defined in Ref.^23^. In the SBT model of the PSC^34,35^, *R*_PSC_/*R*_n_ = 2 $\Lambda_{{\text{Q}}}$/*L*, where *L* is the length of scNW and $\Lambda_{{\text{Q}}}$ is quasiparticle diffusion length. As high-frequency MWs are applied, $\Lambda_{{\text{Q}}}$ becomes longer, which is consistent with the previous study^23^ and gives the value of $\Lambda_{{\text{Q}}}$ = 60–120 nm for the LscNW (*L* = 3.1 μm). In Ref.^23^, on the other hand, there has been a proposal that a naïve SBT model does not apply in this setting as commented above.

**Figure 4:**
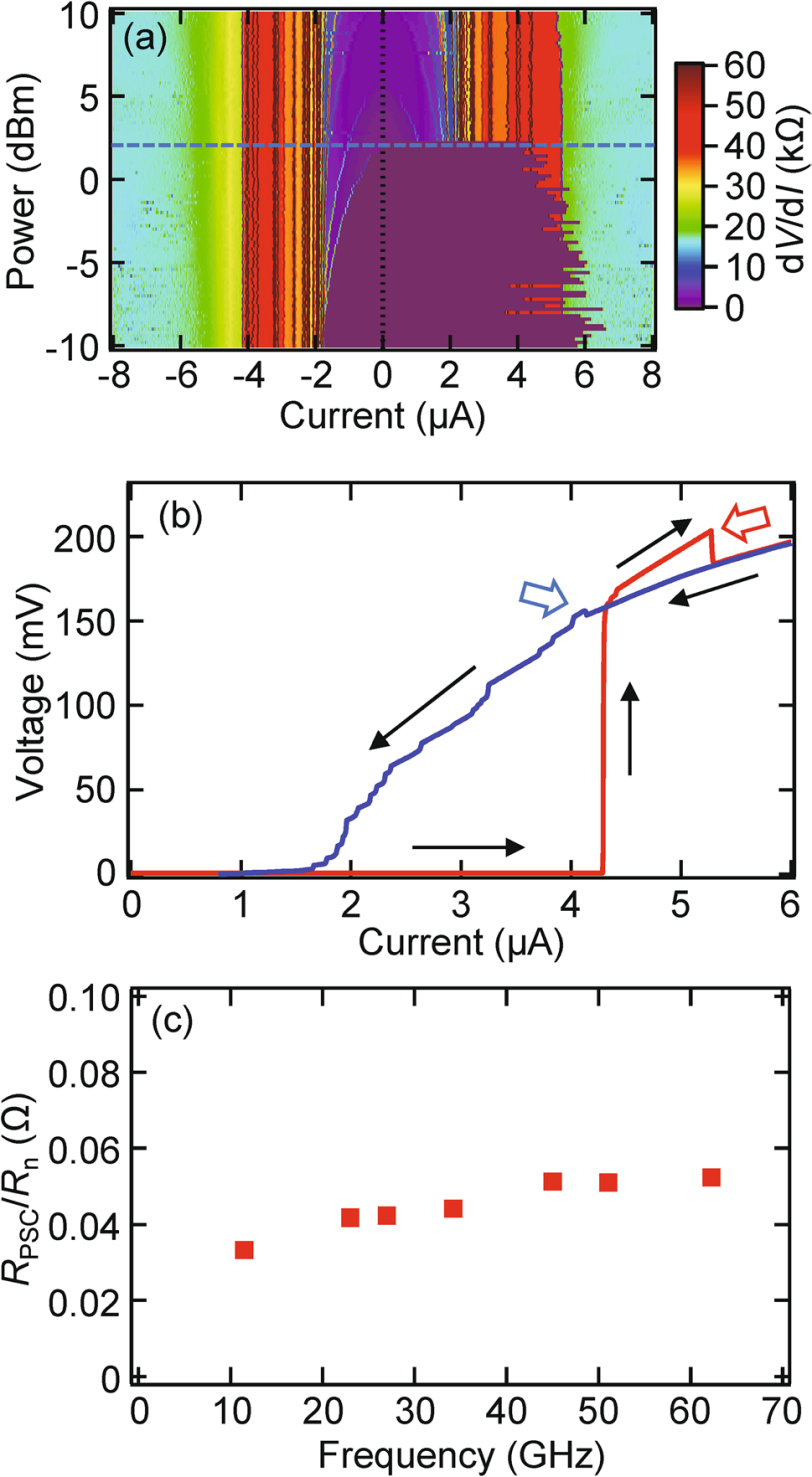
PSC control by MW irradiation in LscNW. (**a**) Mapping of d*V*/d*I* for LscNW as a function of the bias current *I* and MW power *P* at *f* = 26.57 GHz and *T* = 1.65 K. The blue broken line indicates MW power of *P* = 2.2 dBm used in (**b**). (**b**) *I*–*V* characteristics for LscNW at *P* = 2.2 dBm indicated by the blue broken line in (**a**). Open arrows show excess voltages. (**c**) Frequency dependence of *R*_PSC_ (differential resistance of the PSC slightly above *P*_c_, as defined in Ref.^23^).

Now we focus on thinner and short scNW (TscNW). Figure 5a,b show SEM images of TscNW (*L* = 0.75 μm and *W* = 26 nm) and the superconducting film used for a reference, respectively. In this thinner device, PSs become evident away from the critical-fluctuation regime below *T*_c_. Even though thermally activated PS (tPS) dominates near *T*_c_, qPS should play a role at lower temperatures (Fig. 5). These tPS and qPS lead to different temperature dependences. To explain this behavior, we applied the Golubev–Zaikin model^15–19,29^, which describes the qPS contribution to the residual resistance in scNW:

3 $$\begin{aligned} & R_{{{\text{qPS}}}} \left( T \right) = BR_{q} S_{{{\text{qPS}}}} \frac{L}{\xi \left( T \right)}\exp \left( { - S_{{{\text{qPS}}}} } \right) \\ & S_{{{\text{qPS}}}} = C\frac{{R_{{\text{q}}} }}{{R_{{\text{n}}} }}\frac{L}{\xi \left( T \right)}, \\ \end{aligned}$$

where $\xi \left( T \right) = 0.907\xi \left( 0 \right)\left( {1 + \left( {1 - 0.25t} \right)\xi \left( 0 \right)/t} \right)^{ - 1/2} \left( {1 - t^{2} } \right)^{ - 1/2}$, with $t = T/T_{{\text{c}}}$, and *B* and *C* are adjustable parameters. The blue curve in Fig. 5 represents the fit of (3) with $L = 0.9\; {\upmu}\text{m},{ }R_{{\text{n}}} = 7.2\; {\text{k}\Omega},{ }T_{{\text{c}}} = 11\; {\text{K}},$ and $\xi \left( 0 \right) = 3.7\; {\text{nm}}$ and the two adjustable parameters with values of $B = 0.0019$ and $C = 0.014$. Here we note that the two adjustable parameters are smaller than a naïve expectation, which is comparable with the result in Ref.^5^. The red and green curves denote the tPS *R*_tPS_ and total resistance *R*_tot_, respectively^19^. Note that the slight bump/dip structure in the *R*–*T* curve near 8 K is caused by the change in resistance because of the superconducting transition in the film/electrode, which should be because of a shorter sample than that in Ref.^29^. As discussed in Ref.^29^, the APE should also play a role here^29,39–42^. At ~ 8 K, the film, i.e., the electrode, undergoes a phase transition to the superconducting phase where dissipation in the electrode environment is suppressed (Fig. 5b and the black dotted curve in Fig. 5c). The dissipation in this environment, which is coupled to phase slip events, causes the (de)localization of quantum vortices via the Caldeira–Leggett mechanism and APE^43^.

In the above, our discussion is based on the ‘phase-slip’ scenario. Here let us comment on another scenario ‘hot-spot’ scenario. The principle of superconducting single photon detectors is conventionally based on the hot spot and phase slips become unstable in such regime^44,45^. In previous study^5^ on NbN scNW as in our devices, on the other hand, such regime does not take place and phase slips were still stable there. Therefore, as discussed above, consistent picture is given by the ‘phase-slip’ scenario for our devices.

**Figure 5:**
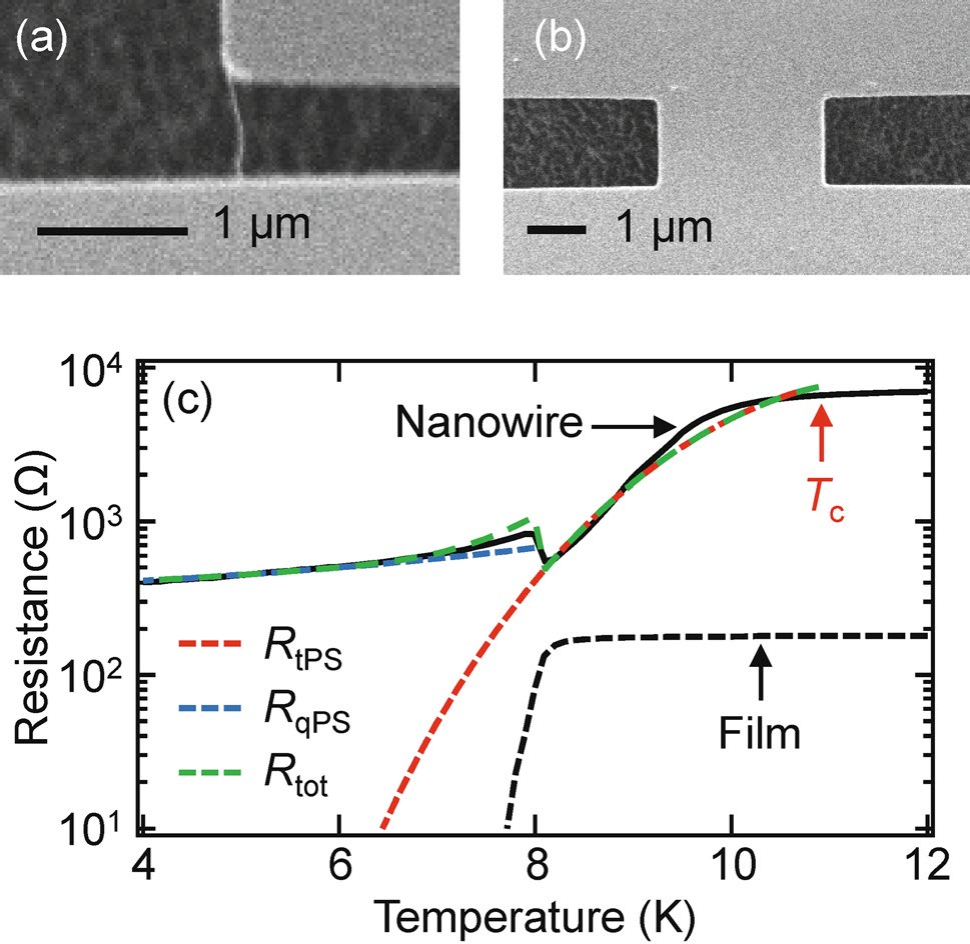
tPS and qPS in TscNW. SEM images of (**a**) TscNW (*L* = 0.75 μm and *W* = 26 nm), and (**b**) superconducting film used for a reference. (**c**) Experimental *R*-*T* characteristic curves (black curve) for TscNW without MW radiation. Red and blue curves are fitting results of two (tPS and qPS) components, respectively. The red and blue broken curves show the tPS/qPS components (*R*_tPS_ and *R*_qPS_, respectively). The green broken curve denotes the total resistance *R*_tot_ of *R*_tPS_ and *R*_qPS_. The black broken curve is the experimental *R–T* for the superconducting film for a reference.

In conclusion, we have studied the superconducting properties of ultrathin nanowires on suspended CNTs. We revealed for the first time the emergence/manipulation of PSs in CNT-templated NbN-scNWs under MW radiation. Dynamical resistive states, i.e., PS centers, are observed via the application of MWs.

## Supplementary information

Supplementary Information.
