# Supplementary material for: Manipulation of phase slips in carbon-nanotube-templated niobium-nitride superconducting nanowires under microwave radiation
Source: Sci Rep. 2020 Aug 31;10:14278. doi: 10.1038/s41598-020-71218-0 (PMC7459120; doi:10.1038/s41598-020-71218-0)
Supplement: Supplementary file 1 — Supplementary Information. [file 41598_2020_71218_MOESM1_ESM.pdf]

Manipulation of phase slips in carbon-nanotube-templated niobium-nitride superconducting nanowires under microwave radiation: Supplemental Material

Kota Kato,[1] Tasuku Takagi,[1] Takasumi Tanabe,[2] Satoshi Moriyama,[3,*] Yoshifumi Morita,[4,†] and Hideyuki Maki[1,5,‡]

[1]*Department of Applied Physics and Physico-Informatics, Faculty of Science and Technology, Keio University, Hiyoshi, Yokohama 223-8522, Japan*

[2]*Department of Electronics and Electrical Engineering, Faculty of Science and Technology, Keio University, Hiyoshi, Yokohama 223-8522, Japan*

[3]*International Center for Materials Nanoarchitectonics (WPI-MANA), National Institute for Materials Science (NIMS), Namiki, Tsukuba, Ibaraki 305-0044, Japan*

[4]*Faculty of Engineering, Gunma University, Kiryu, Gunma 376-8515, Japan*

[5]*Center for Spintronics Research Network, Keio University, Yokohama 223-8522, Japan*

Corresponding authors:

[*] Electronic mail: MORIYAMA.Satoshi@nims.go.jp

[†] Electronic mail: morita@gunma-u.ac.jp

[‡] Electronic mail: maki@appi.keio.ac.jp

The schematics of our experiment setup are shown in Fig. S1. All our measurements were conducted in a pumped helium cryostat. The samples are current-biased in a four-probe configuration. In the current-voltage (*I*-*V*) characteristics measurement in Fig. S1(a), the bias current is applied using a DC source connected in series with a standard resistor whose value is much larger than the sample resistance. The bias current and the voltage across the sample are amplified using either the current or the voltage preamplifier. The outputs of these preamps are fed to the digital multimeters. The microwaves (MWs) are transmitted by a coaxial cable and weakly coupled to the device (see Fig. 1(a)). Resistance-temperature (*R*-*T*) characteristics are obtained using a RF lock-in amplifier in Fig. S1(b).

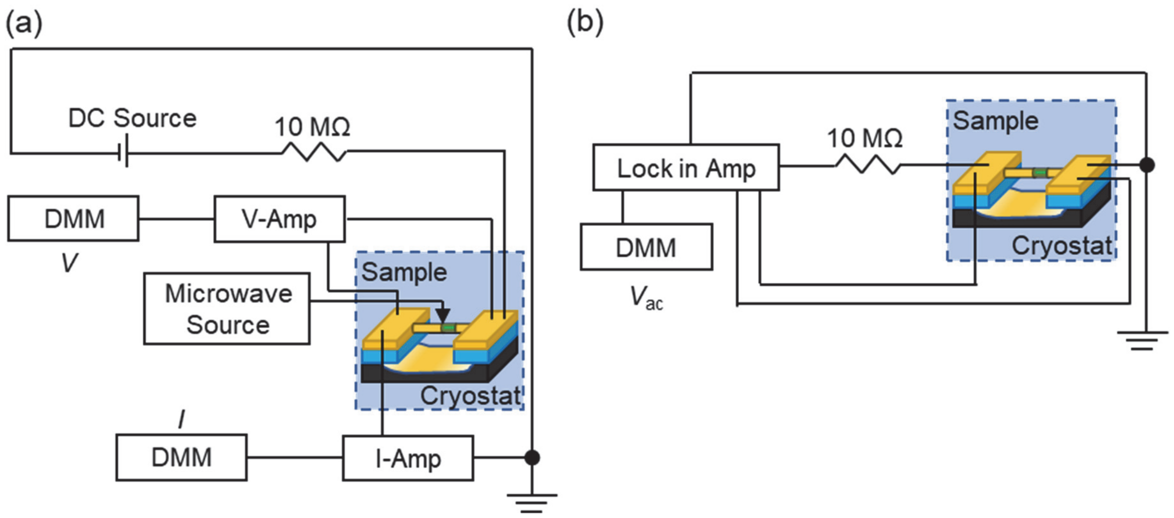

FIG. S1. Schematics of our experimental setting for (a) *I*-*V* characteristics and (b) *R*-*T* characteristics measurements.

In Fig. S2, the mapping of dV/dI is shown for the SscNW as a function of the bias current I and the MW power P at temperature T = 1.65 K with the varied MW frequency.

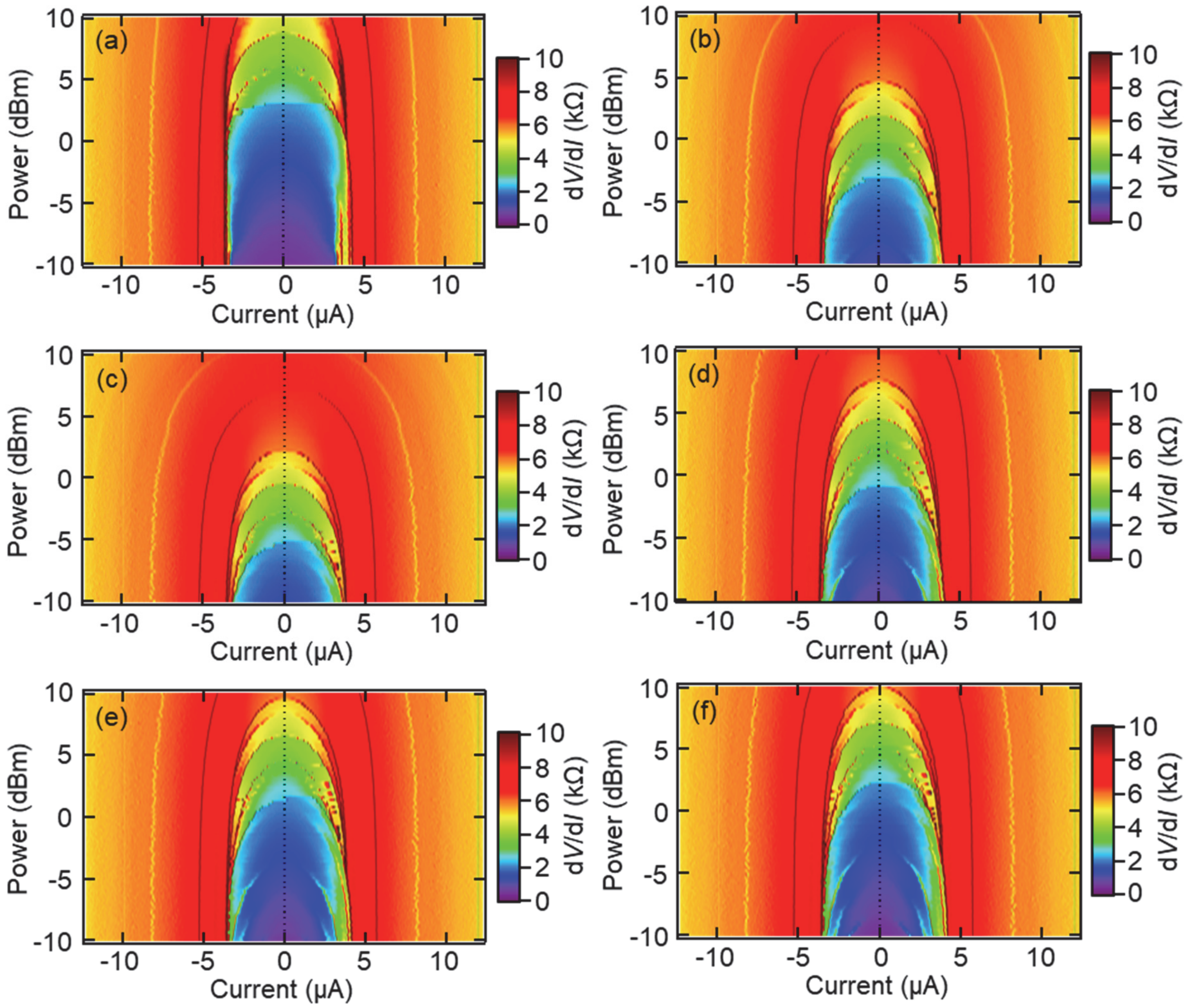

FIG. S2. Mapping of dV/dI for the SscNW as a function of the bias current I and the MW power P at temperature T = 1.65 K. The MW frequencies are (a) 11.06, (b) 22.58, (c) 33.79, (d) 44.60, (e) 50.66, and (f) 61.82 GHz.

In Fig. S3, the mapping of dV/dI is shown for the LscNW as a function of the bias current I and the MW power P at temperature T = 1.65 K with the MW frequency varied. No significant change is observed with varying MW frequency.

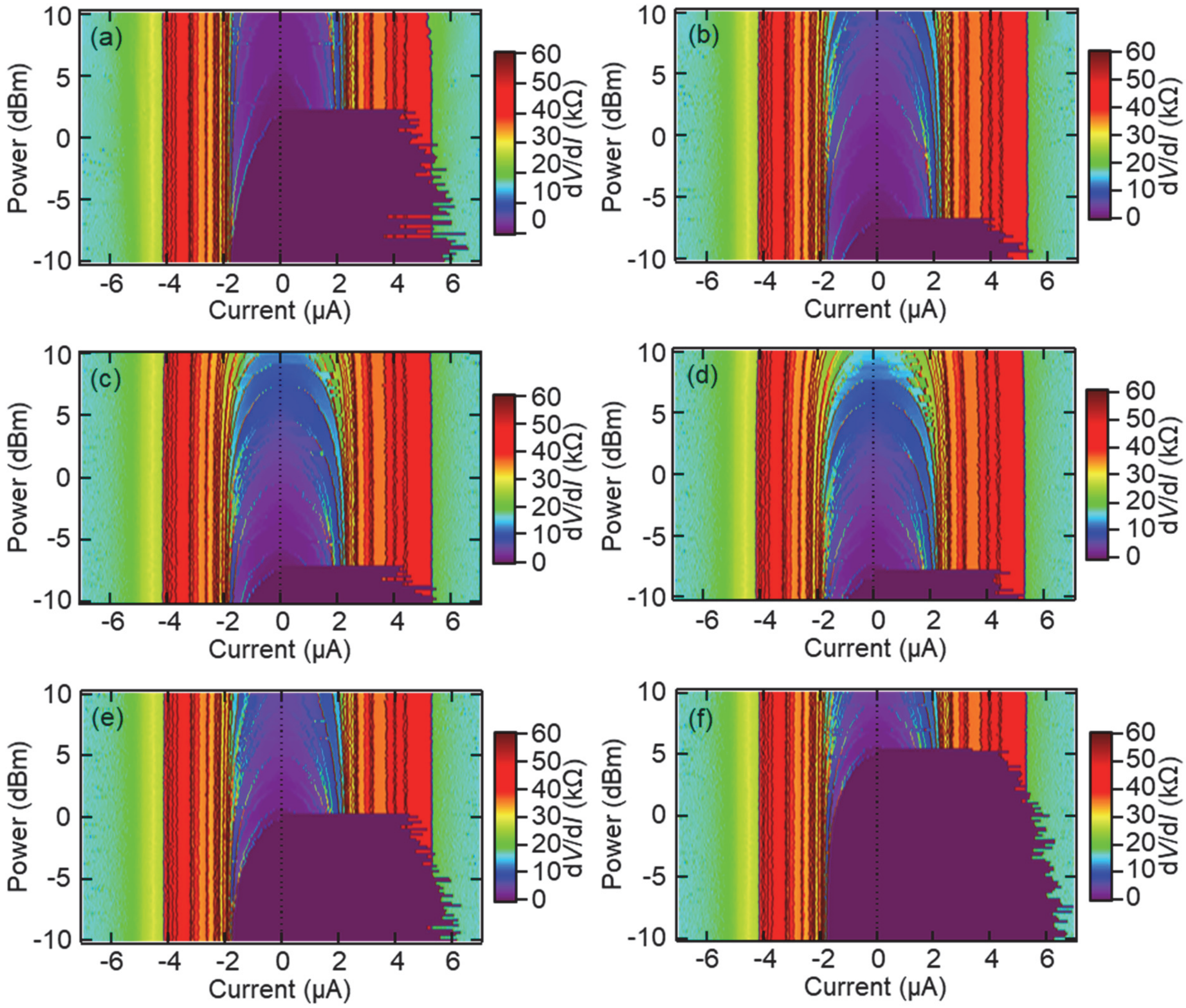

FIG. S3. Mapping of dV/dI for the LscNW as a function of the bias current I and the MW power P at temperature T = 1.65 K. The MW frequencies are (a) 11.06, (b) 22.58, (c) 33.79, (d) 44.60, (e) 50.66, and (f) 61.82 GHz.
